# Timing of menstrual cups to prevent transition from optimal to not optimal vaginal microbiome community state type: Results from a 6.5-year prospective observational cohort

**DOI:** 10.21203/rs.3.rs-10177740/v1

**Published:** 2026-06-30

**Authors:** Supriya D. Mehta, Garazi Zulaika, Souvik Paul, Walter Agingu, Ezekiel Dibondo, Johanna B. Holm, Stefan J. Green, Anna Maria van Eijk, Sanjib Basu, Fredrick Otieno, Penelope A. Phillips-Howard

**Affiliations:** 1 Division of Infectious Disease Medicine, Rush University College of Medicine, 1750 W. Harrison Street, Jelke 1121; Chicago, USA; 2 Division of Epidemiology & Biostatistics, University of Illinois Chicago School of Public Health, 1603 W. Taylor Street, 958; Chicago, USA; 3 Department of Clinical Sciences, Liverpool School of Tropical Medicine, Pembroke Place, Liverpool L3 5QA, UK, Liverpool, UK; 4 Nyanza Reproductive Health Society, UNIM Research and Training Centre off Stadium Road at Lumumba Sub-County Hospital; Kisumu, Nyanza Province, 40100, Kenya; 5 Center for Advanced Microbiome Research and Innovation, Institute for Genome Sciences, Department of Microbiology and Immunology, University of Maryland School of Medicine, 670 W. Baltimore Street, Rm 3117; University of Maryland Baltimore, School of Medicine; Baltimore, MD, 21201, USA; 6 Genomics & Microbiome Core Facility, Rush University Medical Center, 1750 W. Harrison Street, Jelke 444; Chicago, USA

**Keywords:** menstrual health, menstrual cups, vaginal microbiome, *Lactobacillus*, community state type, Markov model, sexually transmitted infection, HSV-2, Bacterial vaginosis

## Abstract

**Background:**

In a cluster randomized trial among Kenyan secondary schoolgirls, menstrual cups were associated with reduced bacterial vaginosis (BV) and increased optimal vaginal microbiome (VMB) community state type (CST-I) prevalence. The prevalence of CST-I decreased over time, suggesting non-optimal VMB is acquired. Using 6.5 years follow-up, we identified how menstrual cups and other factors drive CST transition.

**Methods:**

Participants were randomized to receive menstrual cups (intervention) or standard menstrual management (control). VMB and BV were measured 6-monthly and sexually transmitted infections (STIs; gonorrhea, chlamydia, trichomoniasis) and HSV-2 annually. Control participants received menstrual cups after the 30-month study visit. Continuous-time multi-state Markov modeling estimated the probability of CST transition, focusing on optimal CST-I (*L. crispatus* dominant), CST-III (*L. iners* dominant), and CST-IV (mixed, non-optimal), and identified associated factors.

**Findings:**

Over 6.5 years, in 4,446 observations among 436 participants, CST-I prevalence decreased from 43.3% at baseline to 13.9%, and CST-IV increased from 18.1% at baseline to 44.6%. CST-I and CST-IV were more stable than CST-III. The probability of transition to CST-I from CST-III (16%) or CST-IV (9%) was infrequent. Participants initially randomized to menstrual cups had 54% reduced risk of transitioning from CST-I to CST-IV, adjusted for demographic and behavioral practices, BV, STIs, and HSV-2. Those who were poorer, older, sexually active, and HSV-2 seropositive were more likely to transition to less optimal CSTs.

**Interpretation:**

Over 6.5 years of observation, menstrual cups helped maintain CST-I VMB but did not demonstrate any therapeutic effect and should therefore be given to girls prior to sexual exposure to maximize the likelihood of achieving this benefit.

## Introduction

A non-optimal vaginal microbiome (VMB) and bacterial vaginosis (BV) are associated with increased risk of HIV,^1^ sexually transmitted infections (STIs),^2, 3^ and adverse maternal^4, 5^ and neonatal outcomes.^4–6^ However, interventions to durably improve the VMB and prevent BV recurrence remain limited and with variable efficacy. BV is typically treated with antibiotics, yet recurs in up to 50–80% of women within 3–6 months of treatment.^7^ Results of a randomized trial published in 2025 demonstrated male sex partner treatment improved antibiotic treatment efficacy by 40–45%, though up to 35% of women still experienced recurrence despite partner treatment.^8^ There are mixed findings in relation to probiotics and benefits for VMB and BV due to heterogeneity in formulations and regimens.^9^ Few rigorously conducted randomized controlled trials of live biotherapeutics exist,^10, 11^ and such interventions are still in developmental phases. Given limited treatment options, it is critical to understand factors precipitating non-optimal VMB and BV, to identify behavioral and biological interventions that prevent transition to a non-optimal state.

In our cohort nested within a cluster randomized controlled trial, menstrual cups resulted in a 24% reduced odds of BV and 37% increased odds of an optimal VMB (*Lactobacillus crispatus* dominated) for secondary schoolgirls randomized to receive menstrual cups, as compared to standard practice for menstrual hygiene management (MHM).^12^ Optimal community state type (CST-I; *L. crispatus* dominated) was present in 43% of girls at baseline, the most common CST observed, followed by CST-III (*L. iners* dominated) in 37%, and non-optimal CST-IV (*Gardnerella vaginalis* dominant) being the least common (20%).^13^ However, by the 30-month follow-up visit, the proportion of participants with CST-I had declined to 32%, and CST-IV was observed among 33% of participants; CST-III (*L. iners* dominated) was observed in 35–39% of participants, with no temporal trend.^12^ In extended follow-up, in the cohort overall, the prevalence of BV increased from 12.1% at baseline to 28.1% by 48 months.^14^

The predominance of CST-I at baseline among adolescent girls and young women (AGYW) in our cohort was substantially higher than reported among adult women in sub-Saharan Africa,^13^ and the decline over time in CST-I alongside increased BV and CST-IV is evidence that non-optimal VMB is an acquired state. Our baseline and longitudinal analyses of BV indicated this was correlated with sexual activity. With the completion of 78-months follow-up of this cohort, we sought to quantify transitions in vaginal CSTs associated with menstrual cup use, and identify sociodemographic, behavioral, and biological factors associated with CST transition. Based on our prior analyses, we hypothesized that menstrual cups would be associated with reduced likelihood of transition from CST-I to CST-III and CST-IV, and non-optimal transitions would be more likely with sexual exposure. Identifying key inflection points could inform the optimal timing of menstrual cups interventions to preserve optimal VMB.

## Methods

This study was approved by the institutional review boards of the Rush University (22011311), University of Illinois at Chicago (2017–1301), Liverpool School of Tropical Medicine (15–005), Maseno University Ethics Review Committee (MSU/DRPI/MUERC/01021/21), and the Kenya Medical Research Institutes Scientific Ethics Review Unit (#3215), Written informed consent was obtained for all participants, with written assent and guardian consent obtained for non-emancipated minors. After completion of the trial period (through 30 months), written informed consent was obtained anew for extended follow-up.

### Study Design

Data for this analysis are from Cups and Community Health (CaCHe), a longitudinal cohort study of secondary schoolgirls in western Kenya.^12^ The CaCHe cohort is nested within Cups or Cash for Girls (CCG), initially a cluster randomized controlled trial evaluating the impact of menstrual cups and cash transfer interventions on the composite outcome of school dropout, HIV, and HSV-2.^15^ In the CCG trial, schools (clusters) were randomized (1:1:1:1) to one of four arms: provision of menstrual cups with education on use and care, conditional cash transfer for >80% school attendance per term, menstrual cup plus cash transfer, or standard practice. CaCHe recruited 20% of the CCG trial participants from the cup only and control arms. Eligibility criteria for both CCG and CaCHe included: attendance at participating school, resident of study area, participant assent, consent of parent/guardian, and post-menarche (>3 menstrual bleeds). Girls pregnant at the baseline assessment were excluded. The trial period extended through the 30-month visit, and menstrual cups were provided to all control arm participants after this. Study visits took place at baseline (May-June 2018) and every 6-months following, through 78 months post baseline. The COVID-19 pandemic precluded the 24-month study visit (planned May-June 2020), and the 42-month study visit was missed due to a gap in funding.

### Data Collection

At each study visit, participants self-completed a tablet-based survey in their preferred language (English or DhoLuo) that collected information on socio-demographic characteristics, sexual and MHM practices, and psychosocial variables. Socioeconomic status (SES) was estimated via a household amenity score that considered infrastructure (e.g., water source, light source, sources of heat for cooking, latrine type, flooring and roofing construction) and material possessions (e.g., television, telephone), as previously described.^12^ SES was dichotomized as lower two quintiles vs upper three quintiles for analyses. Time-varying sexual practices questions included: engaging in sexual activity, currently having a main partner/boyfriend/husband, transactional sex, and coerced sex. Other factors included current reported pregnancy, type of hormonal contraceptive use, and any antibiotic use in the past 30 days.

At baseline and alternating visits, participants were tested for HIV and STIs. As reported previously,^12^ two self-collected vaginal swabs were tested for STIs: one for chlamydia and gonorrhea via GeneXpert nucleic acid amplification test (Cepheid, Sunnydale, California, United State), and one for trichomoniasis via rapid immunochromatographic assay (OSOM, Sekisui, Lexington, MA, US). At baseline and every 6 months, participants were tested for BV and VMB was assessed. BV was assessed from a Gram stained slide prepared from a separate self-collected vaginal swab, with Nugent score 7–10 defining BV.^16^ Participants testing positive for BV or STIs were provided antibiotic treatment following Kenyan national guidelines. From a separate self-collected vaginal swab, the V3-V4 region of the bacterial 16S rRNA gene was amplified to characterize the VMB.^12^ Taxonomy was assigned to ASVs using speciateIT.^17^ CSTs were assigned using VALENCIA,^18^ and categorized as: CST-I (*L. crispatus* dominant), -II (*L. jensenii* dominant), or -V (*L. gasseri* dominant), CST-III (*L. iners* dominant), or CST-IV (non-optimal). Raw sequence data (FASTQ files) were deposited in the National Center for Biotechnology Information (NCBI) Sequence Read Archive (SRA), under BioProject identifier PRJNA746243.

### Statistical Analyses

**Figure 1.**
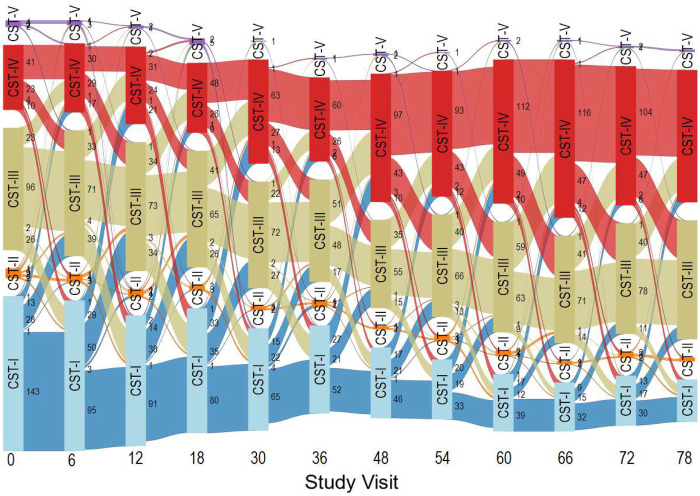
Change in community state types over time The Sankey diagram shows the transitions between CST-I (blue), -II (orange), -III (khaki), -IV (red), -V (purple) over time. The numbers inside or to the side of the flows represent the number of observations remaining in the state or changing state.

We applied a continuous-time multi-state Markov model to estimate the probability of transitioning from one CST to another,^19, 20^ focusing on transitions among CST-I, -III, and -IV. CST-II and CST-V were excluded from analyses due to sparsity (Table 1, Figure 1). This modeling approach is particularly well-suited for capturing dynamic processes in longitudinal settings.^21, 22^ In our analysis, we investigated the influence of intervention states, age at baseline, SES, sexual exposures, antibiotic use, hormonal contraceptive use, STI and HSV-2 status, as these have been previously shown to impact VMB.^23, 24^ Both unadjusted and covariate-adjusted hazard ratios (HRs) were estimated. We also performed stratified analyses to explore potential differential effects of menstrual cups across subgroups defined by BV status, STI status, HSV-2 status, sexual activity, and age at sexual debut (<17 vs. ≥17). To ensure model stability in some stratum-specific adjusted analyses, a reduced subset of the covariates was adjusted for, and CST transitions were constrained to share the same intensities for selected strata (Supplemental Methods). Each Markov model estimated transition probabilities per unit time (6 months) between CSTs. Model fitting was conducted using the msm package in R (version 4.5.1).^20^

To aid inference, we conducted analyses to identify factors associated with current menstrual cup use (i.e., reporting menstrual cup use at the last menstrual period), stratified by initial randomization status. We *a priori* hypothesized that factors associated with menstrual cup use would include age and socioeconomic status. Because participants knew the study was assessing the potential effect of menstrual cups on BV and STIs, we also hypothesized that sexual exposures, BV, STI, or HSV-2 status might also affect menstrual cup uptake. For the control arm model, factors associated with menstrual cup use were modeled from the 36-month period onwards, since cups were distributed after the 30-month study visit. In models for control arm, we did not test baseline characteristics due to temporal remoteness (3 years in the past), and evaluated only time-varying covariates, and age at the 30-month visit. Generalized linear mixed models (GLMMs) were fitted to allow for the hierarchical structure of the data. Models with a Poisson distribution and log link included participant and cluster as random effects, and estimated prevalence ratios and 95% confidence intervals across all time points, with robust variance estimation.

### Missing data

Most missing CST values (59 of 87, 68%) were from the 36-month visit, when phone interviews were conducted among participants who had relocated. Additionally, 59 participant surveys were missing from the 6-month visit due to a loss in server access. We performed complete case analyses.

### Power and Sample Size

The sample size for the trial phase of CaCHe (220 in the cup arm and 220 in the control arm) was calculated to detect hypothesized difference in the prevalence of BV between intervention and control arm during the trial, accounting for repeated measures and loss to follow-up;^12^ no further sample size or power calculations were conducted for the period of extended follow-up.

### Role of the funding source

The funder of the study had no role in study design, data collection, data analysis, data interpretation, or writing of the report.

## Results

Over time, the proportion of participants reporting themselves to be sexually active increased from 34.8% at baseline to 90.2% at 78 months (Table 1). As has been reported previously for this cohort,^25^ pregnancy and contraceptive use were infrequent prior to 30 months study follow-up, and increased substantially thereafter. Despite high antibiotic treatment rates for BV and STIs, the prevalence of BV and STIs increased over time, as did the prevalences of HSV-2, and HIV also increased over time. During the trial, follow-up was maintained at ≥90% (Table 1). After the 30-month visit, participant follow-up ranged from a low of 83.5% (48- and 54- month visits) to 90.1% (60-month visit), and 78.5% of participants had at least ten VMB measurements.

### Descriptive Trends in Vaginal Community State Types over Time

In keeping with the increase in BV over the 6.5-year period, the prevalence of CST-I decreased from 43.3% at baseline to 13.9%, and CST-IV prevalence increased from 18.1% at baseline to 44.6% (Table 1, Figure 1).

### Probability of Transition in Community State Types

**Figure 2.**
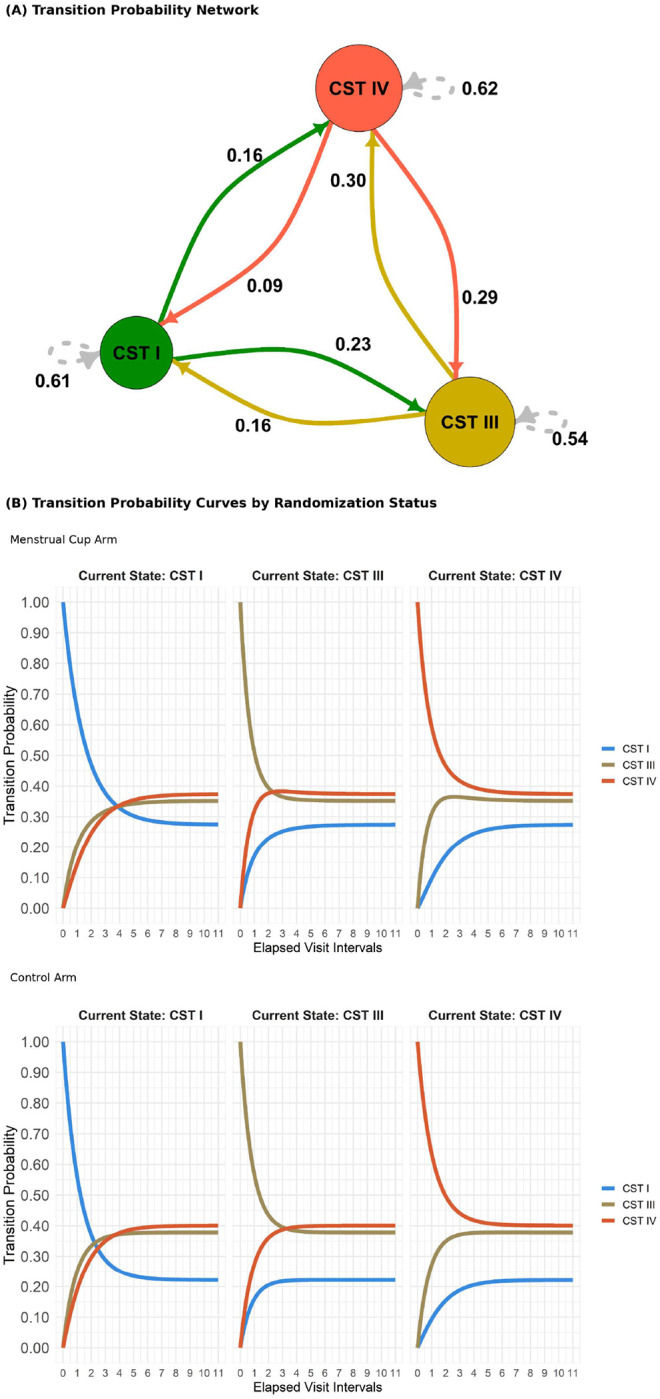
Transition probability network diagram of CSTs and Transition Probability Curves (A) Dynamics of the vaginal communities were approximated as a Markov State with subject-independent transition probabilities between CSTs. (B) Transition probability curves showing the probability of transitioning between states stratified by starting state (CST-I, CST-III, or CST-IV), stratified by intervention status (menstrual cup arm, control arm). For each current state (CST-I, CST-III, or CST-IV), at time zero their probability begins at 1.0 because that is the state the participant starts in.

Figure 2A displays the estimated per-visit transition probabilities per 6-month interval between CST-I, -III, and -IV obtained from the multi-state Markov model. Node sizes are proportional to the overall prevalence of each CST, while directed edges represent transition probability per unit visit between states, with self-loops (dotted) indicating the same of remaining in the same state. CST-I and CST-IV were more stable, with the probability of remaining in the state of 0.61 and 0.62, respectively, as compared to CST-III (0.53). The probability of transition from CST-I to a worse state (CST-III 23%, or CST-IV 17%) was greater than the probability of transition to CST-I from CST-III (16%) or CST-IV (9%).

Figure 2B shows the probability of being in each CST over time depending on the initial CST, stratified by menstrual cup arm and control arm. For participants beginning in CST-I, the probability of remaining in CST-I declined over time (blue line), while the probability of CST-IV (red) and CST-III (olive) increased, reflecting the increasing probability of transitioning to these states. For both intervention arm and control arm participants, long-term equilibrium was reached after approximately 4- to 6- time intervals (i.e., the lines representing probability of change flattened). However, the stationary distribution differed by intervention and control arm. Among control participants, the long-run equilibrium settled at CST-I = 22.3%, CST-III = 37.7%, CST-IV = 39.9%, compared to CST-I = 27.6%, CST-III = 34.9%, CST-IV = 37.2% among intervention participants. This represents an increase in the proportion of time spent in optimal CST-I, and a corresponding reduction in time spent in non-optimal states.

### Factors associated with transition in Community State Types

In multivariable analyses according to randomization status (Table 2), participants in the menstrual cup arm had a 54% reduced risk of transitioning from CST-I to CST-IV (adjusted hazard ratio [aHR] = 0.46; 95% CI: 0.27 – 0.77). Cup intervention arm participants were more likely to transition from CST-III to CST-IV and from CST-IV to CST-III, reflecting the relatively less stable nature of these states and the convex nature of CSTs. Other multivariable adjusted analyses indicated that participants were more likely to transition from CST-I to CST-III with increasing age; participants with lower SES had more than 2-fold risk of transitioning from CST-I to CST-IV; sexually active participants were more likely to transition from CST-III to CST-IV, and HSV-2 seropositivity was associated with more than 4-fold risk of transitioning from CST-I to CST-IV. Recent antibiotic use and age at sexual debut were not statistically significantly associated with any transitions. There were no factors associated with transition from CST-IV to CST-I or CST-III to CST-I.

Pregnancy and hormonal contraceptive use were sparse up to the 30- and 36- month study visits (Supplemental Table 1). While some unstable estimates are produced, participants using implant contraceptives (aHR = 5.50), were more likely to transition from CST-I to CST-IV, and participants reporting injectable contraceptive use were more likely to transition from CST-I to CST-III (aHR = 3.11), adjusted for intervention status, age at baseline, age at sexual debut, and time-varying covariates - SES, sexual activity, recent antibiotic use, and HSV-2. Pregnant participants were more likely to transition from CST-III to CST-IV (aHR = 2.15), and HIV infected participants were 4.5 times more likely to transition from CST-I to CST-IV, though this did not remain statistically significant in a fully adjusted model.

### Stratum Specific Analyses

The preventive effects on transition from CST-I to CST-IV associated with being in the original menstrual cup arm were observed primarily for AGYW that were BV negative or HSV-2 negative at baseline (Table 3). In crude analyses, preventive effects on transition from CST-I to CST-IV were observed among participants who were not sexually active at baseline (aHR=0.47) more so than among those who were sexually active (aHR=0.68), but effects were attenuated and non-significant when adjusted for age at baseline, time-varying SES, time-varying HSV-2 status, and age at sexual debut. Similarly, preventive effects among those who were STI negative at baseline were attenuated when adjusted for age at baseline, time-varying SES, time-varying HSV-2 status, age at sexual debut, and recent antibiotic use.

### Menstrual Cup Use Over Time and Associated Factors

**Figure 3.**
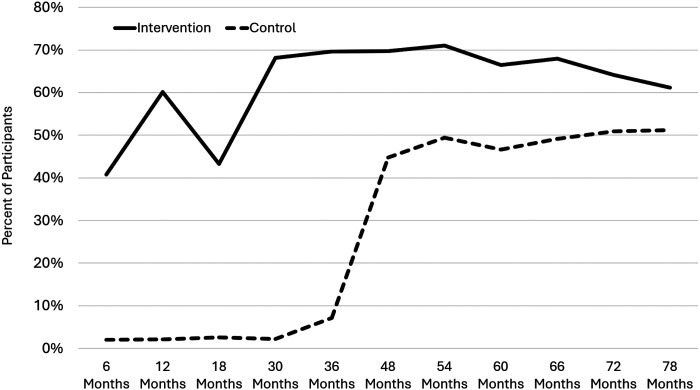
Percent of participants reporting menstrual cup use at last menses, stratified by randomization status The percent of participants reporting menstrual cup status (y-axis) over time (x-axis) is shown for participants randomized to Intervention (solid line) and Control (dashed line). The decline in menstrual cup use at the 18-month visit for Intervention arm participants was due to a survey error that inadvertently excluded questions about menstrual cup use.

Menstrual cups were provided to intervention arm participants after the baseline visit and to the control arm participants after the 30-month visit (Figure 3). Among intervention participants, uptake rose from approximately 40% at the 6-month visit to approximately 70% by 30-months. Among control arm participants, menstrual cup use rose to approximately 50% by the 54-month visit and remained relatively stable through 78-months.

**Figure 4.**
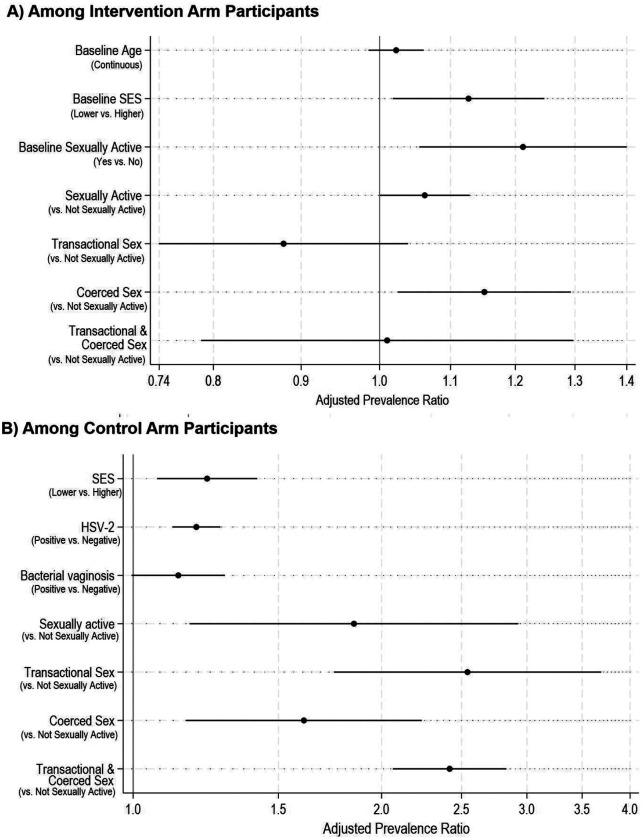
Factors associated with current menstrual cup use, stratified by randomization status The coefficient plot shows the adjusted prevalence ratio (black circle) and 95% confidence interval (black horizontal line), quantified on the x-axis, with factor specified on the y-axis.

We examined factors associated with menstrual cup use to understand biases or confounding that potentially may be introduced in an analysis examining cup use rather than randomization status in relation to VMB. In multivariable mixed effects modeling, among intervention arm participants (Figure 4A, and Supplemental Table 2), menstrual cup use was more likely to be reported among those with lower SES and sexual activity at baseline or over follow-up. Among control participants (Figure 4B, and Supplemental Table 2), menstrual cup use was more likely to be reported by those diagnosed with an STI at baseline, and several time-varying factors: lower SES, infection with HSV-2 or BV, and being sexually active, including highest menstrual cup use among AGYW reporting transactional sex or coerced sex.

### Distribution of CST-I over Time by Randomization Status, Cup Use, and Sexual Activity

**Figure 5.**
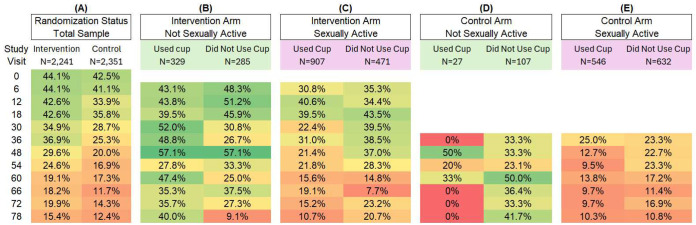
Prevalence of Community State Type I (*L. crispatus* dominant) by study visit, overall and stratified by randomization arms (intervention or control), sexual activity, and reported cup use To facilitate visual trend detection, higher proportion of CST-I is shaded green, intermediate values yellow, and lower values orange and red.

While both arms had similar prevalence of CST-I at baseline and at 78-months, the decline occurred earlier for control arm participants, and the prevalence of CST-I remained higher for intervention arm participants (Figure 5A). As shown in Figure 5B, the prevalence of CST-I remained higher among participants randomized to intervention who reported cup use and were not sexually active. Participants randomized to intervention who were sexually active cup users (Figure 5C) had generally lower prevalence of CST-I compared to non-users, as those with greater sexual exposure were more likely to use cups. Figures 5D and 5E show the prevalence of CST-I from the 36-month visit onwards among participants randomized to the control arm, stratified by sexual activity and cup use. CST-I is generally lower among participants reporting cup use, and likely due to higher likelihood of cup use among participants with greater probability of sexual exposure and STIs.

## Discussion

In this cohort of Kenyan AGYW followed for 6.5 years, optimal CST-I was the most common vaginal CST at cohort entry (43%) but declined to 13% of participants over follow-up. Transition from CST-I to CST-IV increased with sexual activity and indicators of sexual exposure – BV, STIs, and HSV-2. Being in the menstrual cup arm was protective of transition from CST-I to non-optimal CST-IV. The probability of transitioning from non-optimal CST-IV to optimal CST-I was infrequent (9%) and no factors were identified that increased the likelihood of recovery from a non-optimal to optimal CST. Among control arm participants, at the time menstrual cups were distributed (30-months after baseline, median age 19.4 years), a minority had CST-I, and no benefit to VMB of menstrual cup use was observed.

The probability of transition from CST-I to CST-IV was more than halved among menstrual cup intervention arm participants over the 6.5-year follow-up, with the higher prevalence of CST-I being maintained for at least 5 years. Our analyses show that the benefit of menstrual cups derives from a preventive effect against transitioning to a non-optimal vaginal CST, and that they do not confer a therapeutic effect of transitioning to a “better” CST. This means that menstrual cups could be most beneficial when given to girls with CST-I, which is more likely at younger ages and prior to sexual exposure. When control arm participants were provided menstrual cups after the 30-month visit, participants were median age 19.4 years, twice as many reported sexual activity compared to baseline, the majority did not have CST-I, and cup use was more likely among participants with increased risk of non-optimal CST. Therefore, we did not conduct an as treated analyses (i.e., according to reported cup use) as this would have introduced confounding, due to the overt selection bias among cup users with higher likelihood of sexual exposure, BV, and STIs.

The preventive effect of menstrual cups on the VMB is biologically plausible through the stabilisation of the vaginal environment during menses. Menstruation is associated with transient increases in vaginal pH, iron availability, and microbial diversity, which can favour the growth of anaerobic taxa and destabilise *Lactobacillus*-dominated communities.^26^ The VMB commonly shifts away from optimal states during menses before recovering, or partially recovering, thereafter. One study by Krog and colleagues found that 58% of women had a dysbiotic vaginal microbiome during menses with a statistically significant reduction in dysbiosis to 32% in the follicular phase and 29% in the luteal phase post-menses.^27^ Menstrual cups may mitigate this disruption by containing menstrual blood within the cup chamber and reducing exposure of the vaginal epithelium and microbiome to blood-associated substrates. In this way, cups may act not as a therapeutic agent, but as a buffer against cyclical disruptions that precipitate transition to non-optimal CSTs.

The prevalence of CST-I decreased over time. In adjusted analyses, sexual activity, BV, HSV-2, and HIV were independently associated with transition from an optimal to non-optimal state. This is in keeping with the literature demonstrating the association between these factors and CST.^28–30^ Although HIV status was analyzed as a time-varying covariate, it was measured annually and thus temporality in relation to CST transitions is obscured. Literature on the association between hormonal contraceptive use and VMB is mixed, with oestrogen-containing contraception generally associated with *Lactobacillus*-dominant profiles and reduced BV risk, whereas progestin-only methods show heterogeneous associations, including reports of increased microbial diversity and no association, depending on population and microbiome definition.^31^ In our analysis, there may be time-varying confounding associated with both hormonal contraceptive use and CST transition that is not adequately addressed with multivariable adjustment.

The probability of transition from CST-IV to CST-I was rare. This implies that once the vaginal microbiome loses *Lactobacillus* dominance, in the absence of external therapeutic intervention, the internal dynamics are not likely to shift it back. This occurred irrespective of study arm and despite antibiotic treatment for BV and reproductive tract infections, highlighting the importance of prevention as a primary goal to VMB interventions. Menstrual cups are one such tool that can help support a favorable VMB community before disruption occurs. Complementary strategies should focus on reducing exposure to known risk factors for dysbiosis, including sexual exposures, and targeting transmission dynamics within sexual networks for BV and STIs (e.g., male partner treatment to reduce BV recurrence). Further research is needed that includes male partner penile microbiome studies to identify additional intervention targets and help shift the burden of prevention and treatment beyond women alone.

Menstrual cup use was lower among control arm participants than intervention arm participants. When control participants were given their menstrual cups after the trial ended, many did not receive the menstrual cup training in the same group format as the intervention arm because schools were closed due to the COVID-19 pandemic, and many had graduated, so were often individually trained. Research has shown that group-based interventions can be more effective than individual intervention,^32^ and this may have contributed. Specifically, school-based cohorts may be especially effective, supporting social interaction among peers in relation to the intervention. Additionally, the quality and intensity of the intervention training may have been weakened due to the more fragmented nature of study visits during the COVID-19 pandemic.

### Limitations and Strengths

Study limitations include the following. First, generalisability may be limited to similar rural populations of adolescent girls and young women. Second, substantial missingness in sexual exposure data, including for condom use, partner number, partner type, male circumcision status, and age-disparate relationships, due to underreporting of sexual activity and some survey error, may have resulted in some residual confounding. Cup use was self-reported and participants at higher risk of BV and STIs may have been more likely to report cup use, knowing that the study was about menstrual cup and risk of vaginal infections. VMB assessments were conducted primarily every 6 months, though there were some gaps; however, follow-up was high and the majority of participants had ten or more observations. Unlike standard longitudinal approaches to identifying factors associated with VMB composition, the temporal nature of the Markov model captures the probabilistic nature, directionality, and timing of transitions between microbiome states. This allows identification of inflection points and differentiation between preventive versus restorative effects of exposures, insights that are not obtainable from standard prevalence-based analyses and are directly relevant to informing intervention efficacy and timings.

## Conclusions

Our results indicate that the benefits of menstrual cups lie in helping maintain a CST-I VMB and should therefore be given to AGYW prior to sexual exposure to maximize the likelihood of achieving this benefit. Research in other populations is needed to verify these findings and determine wider relevance.

## Supplementary Material

1

## Figures and Tables

**Table 1.** Distribution of participant characteristics by time point.

	Baseline, N=436 n (%)	6 Months, N=421 n (%)	12 Months, N=384 n (%)	18 Months, N=394 n (%)	30 Months, N=396 n (%)	36 Months, N=384 n (%)	48 Months, N=365 n (%)	54 Months, N=362 n (%)	60 Months, N=391 n (%)	66 Months, N=385 n (%)	72 Months, N=376 n (%)	78 Months, N=369 n (%)
Randomization status												
Intervention	213 (48.9)	202 (48.0)	189 (49.2)	190 (48.2)	194 (49.0)	184 (47.9)	180 (49.3)	172 (47.5)	194 (49.6)	188 (48.8)	186 (49.5)	182 (49.3)
Control	223 (51.1)	219 (52.0)	195 (50.8)	204 (51.8)	202 (51.0)	200 (52.1)	185 (50.7)	190 (52.5)	197 (50.4)	197 (51.2)	190 (50.5)	187 (50.7)
Current cup use	0 (0)	75 (20.1)	117 (30.7)	87 (22.3)	132 (33.5)	144 (37.7)	212 (58.1)	211 (58.3)	218 (55.7)	224 (58.2)	217 (57.7)	214 (58.0)
*Missing*	0	47	3	4	2	2	0	0	0	0	0	0
Community State Type												
CST-I *(L. crispatus)*	187 (43.3)	179 (42.5)	146 (38.1)	154 (39.1)	125 (31.7)	101 (30.9)	90 (24.7)	75 (20.7)	71 (18.1)	57 (15.9)	64 (17.1)	51 (13.9)
CST-II *(L. gasseri)*	12 (2.8)	9 (2.1)	9 (2.4)	6 (1.5)	5 (1.3)	7 (2.1)	4 (1.1)	9 (2.5)	7 (2.0)	4 (1.0)	7 (1.9)	5 (1.4)
CST-III *(L. iners)*	147 (34.0)	149 (35.4)	146 (38.1)	140 (35.5)	136 (34.5)	120 (36.7)	109 (30.0)	124 (34.2)	135 (34.6)	131 (34.2)	133 (35.5)	145 (39.4)
CST-IV (mixed)	78 (18.1)	78 (18.5)	79 (20.6)	86 (21.8)	127 (32.2)	97 (29.7)	158 (43.4)	153 (42.3)	176 (44.8)	189 (49.4)	168 (44.8)	164 (44.6)
CST-V *(L. jensenii)*	8 (1.9)	6 (1.4)	3 (0.8)	8 (2.0)	1 (0.3)	2 (0.6)	3 (0.8)	1 (0.3)	2 (0.5)	2 (0.5)	3 (0.8)	3 (0.8)
*Missing*	4	0	1	0	2	57	1	2	0	2	1	1
BV positive	49 (11.3)	39 (9.3)	56 (14.6)	55 (14.0)	88 (22.3)	71 (23.6)	102 (28.0)	92 (25.6)	113 (29.0)	132 (34.5)	110 (29.3)	122 (33.2)
*Missing*	4	0	0	0	1	83	0	2	1	2	1	2
BV treated	48 (98.0)	37 (94.9)	54 (96.4)	55 (100)	79 (89.8)	66 (93.0)	100 (98.0)	90 (97.8)	105 (92.9)	130 (98.5)	107 (97.3)	118 (96.7)
STI positive	43 (14.4)		42 (10.9)		64 (21.5)		73 (24.5)		72 (24.2)		82 (21.9)	
*Missing*	4		0		1		1		2		1	
STI treated	42 (97.7)		42 (100)		57 (89.1)		67 (91.8)		68 (94.4)		78 (95.1)	
HSV-2 seropositive	61 (14.2)	58 (14.5)	63 (16.5)	63 (17.5)	84 (21.6)	75 (24.4)	76 (25.1)	78 (25.8)	142 (36.4)	139 (40.6)	157 (43.4)	155 (42.5)
*Missing*	6	21	1	33	7	77	62	60	1	43	14	4
HIV positive	7 (1.6)	7 (1.7)	10 (2.5)	10 (2.6)	10 (2.5)	11 (3.0)	12 (3.3)	13 (3.7)	14 (3.6)	14 (3.6)	17 (4.5)	17 (4.6)
*Missing*	2	7	0	8	1	19	1	8	2	22	0	0
Median age (IQR)	16.9 (16.1–17.9)	17.3 (16.4–18.3)	17.9 (17.0–18.8)	18.3 (17.4–19.2)	19.4 (18.5–20.3)	19.9 (19.0–20.8)	20.9 (20.0–21.8)	21.3 (20.5–22.2)	22.0 (21.1–22.9)	22.4 (21.5–23.3)	22.9 (22.0–23.8)	23.4 (22.5–24.3)
Socioeconomic status												
Two lowest quintiles	121 (27.8)	123 (29.2)	116 (30.2)	116 (29.4)	118 (29.8)	98 (25.5)	146 (40.0)	146 (40.3)	160 (40.9)	154 (40.0)	152 (40.4)	157 (42.5)
Three higher quintiles	309 (70.2)	301 (70.8)	268 (69.8)	278 (70.6)	278 (70.2)	228 (59.4)	210 (60.0)	216 (59.7)	231 (59.1)	231 (60.0)	224 (59.6)	212 (57.5)
*Missing*						58						
Sexually active^1^	149 (34.9)	72 (19.3)	211 (55.4)	217 (55.6)	253 (64.2)	275 (72.0)	326 (89.3)	320 (88.4)	349 (89.3)	345 (89.6)	332 (88.3)	333 (90.2)
*Missing*	9	47	3	4	2	2	0	0	0	0	0	0
Transactional sex	51 (11.9)	17 (4.6)	32 (8.4)	32 (8.2)	26 (6.6)	37 (9.7)	33 (9.0)	42 (11.6)	51 (13.0)	62 (16.1)	51 (13.6)	22 (6.0)
*Missing*	9	47	3	4	2	2	0	0	0	0	0	0
Coerced sex	77 (18.0)	23 (6.2)	51 (13.4)	51 (13.1)	40 (10.2)	36 (9.4)	47 (12.9)	44 (12.2)	34 (8.7)	33 (8.6)	26 (6.9)	9 (2.4)
*Missing*	9	40	10	4	2	2	0	0	0	0	0	0
Age at sexual debut <17 (vs. 17 or older)	138 (32.8)	132 (32.1)	120 (32.1)	129 (33.6)	131 (33.8)	125 (33.2)	121 (33.6)	118 (32.9)	129 (33.2)	130 (34.0)	129 (34.6)	126 (34.3)
*Missing*	15	40	10	10	8	7	7	3	2	3	3	2 (0.5)
Has a boyfriend/husband	47 (11.0)	23 (6.2)	83 (21.8)	12 (3.1)	136 (34.5)	168 (44.0)	241 (66.0)	239 (66.0)	250 (63.9)	257 (66.8)	264 (70.2)	263 (71.3)
*Missing*	5	47	3	4	2	2	0	0	0	0	0	0
Currently pregnant	8 (1.9)	7 (1.9)	6 (1.6)	8 (2.1)	36 (9.1)	32 (8.4)	48 (13.2)	38 (10.5)	32 (8.2)	31 (8.1)	32 (8.5)	32 (8.7)
*Missing*	9	47	3	4	2	2	0	0	0	0	0	0
Any condom use among sexually active	110 (85.3)	54 (91.5)	153 (73.9)	165 (76.7)	197 (80.4)	234 (86.4)	268 (82.7)	270 (85.2)	254 (72.8)	234 (67.8)	193 (58.1)	187 (59.0)
*Missing*	20	13	4	2	8	4	2	3	0	0	0	16
Hormonal contraceptive use	11 (2.5)	13 (3.5)	11 (2.8)	10 (2.5)	20 (5.1)	25 (6.5)	67 (18.4)	82 (22.5)	99 (25.2)	117 (30.4)	121 (32.2)	123 (33.3)
Implant	4	6	9	7	14	17	36	44	56	54	65	69
Injection	6	7	1	3	6	8	27	31	31	48	45	48
*Missing*	4	47	3	4	2	2	0	0	0	0	0	0
Antibiotics past 30 days	84 (19.9)	71 (19.0)	86 (22.6)	79 (20.3)	75 (19.0)	79 (20.7)	83 (22.8)	87 (24.0)	130 (33.3)	128 (33.2)	132 (35.1)	112 (30.4)
*Missing*	14	47	3	4	2	2	0	0	0	0	0	0

1 Transactional sex and coerced sex are a subset of sexually active.

**Table 2.** Results of Markov Modeling: Unadjusted and Multivariable Adjusted Hazard Ratios

Variable	Unadjusted HR (95% CI)	Adjusted*, N=4022 HR (95% CI)
Intervention status: Menstrual cups vs. Control	N=4,466	
CST-I to CST-III	0.81 (0.59, 1.13)	0.99 (0.70, 1.40)
CST-I to CST-IV	0.62 (0.37, 1.05)	**0.46 (0.27, 0.77)**
CST-III to CST-I	1.05 (0.75, 1.47)	0.92 (0.65, 1.32)
CST-III to CST-IV	**1.37 (1.06, 1.78)**	**1.61 (1.19, 2.16)**
CST-IV to CST-I	0.82 (0.37, 1.81)	0.99 (0.48, 2.03)
CST-IV to CST-III	**1.33 (1.02, 1.74)**	1.33 (0.98, 1.79)
Baseline Age in Years, Continuous	N=4,424	
CST-I to CST-III	1.12 (0.99, 1.27)	**1.19 (1.03, 1.37)**
CST-I to CST-IV	1.06 (0.87, 1.28)	0.85 (0.68, 1.08)
CST-III to CST-I	0.93 (0.81, 1.07)	0.92 (0.76, 1.13)
CST-III to CST-IV	1.08 (0.97, 1.19)	1.12 (0.97, 1.29)
CST-IV to CST-I	0.94 (0.71, 1.24)	0.85 (0.58, 1.24)
CST-IV to CST-III	0.97 (0.89, 1.07)	0.99 (0.87, 1.13)
Baseline SES (lower two quintiles vs. upper three quintiles)	N=4,466	
CST-I to CST-III	**1.61 (1.14, 2.29)**	1.34 (0.78, 2.29)
CST-I to CST-IV	1.09 (0.60, 1.96)	**2.34 (1.15, 4.76)**
CST-III to CST-I	1.32 (0.92, 1.90)	1.48 (0.82, 2.70)
CST-III to CST-IV	1.01 (0.77, 1.33)	1.09 (0.72, 1.65)
CST-IV to CST-I	0.96 (0.42, 2.20)	2.21 (0.86, 5.70)
CST-IV to CST-III	0.86 (0.65, 1.15)	0.75 (0.52, 1.08)
Sexually Active (yes vs. no)	N=4,406	
CST-I to CST-III	1.05 (0.75, 1.46)	0.92 (0.60, 1.39)
CST-I to CST-IV	**1.90 (1.12, 3.22)**	1.66 (0.90, 3.05)
CST-III to CST-I	**0.68 (0.48, 0.96)**	0.72 (0.49, 1.06)
CST-III to CST-IV	**1.47 (1.09, 1.99)**	**1.43 (1.00, 2.04)**
CST-IV to CST-I	0.46 (0.23, 0.96)	0.55 (0.23, 1.31)
CST-IV to CST-III	1.10 (0.79, 1.53)	1.01 (0.67, 1.54)
HSV-2 positive (vs. negative)	N=4,210	
CST-I to CST-III	0.95 (0.63, 1.45)	1.87 (0.69, 5.10)
CST-I to CST-IV	0.91 (0.48, 1.72)	**4.32 (1.64, 11.4)**
CST-III to CST-I	**0.61 (0.37, 0.99)**	0.62 (0.21, 1.79)
CST-III to CST-IV	1.32 (0.99, 1.77)	0.92 (0.57, 1.50)
CST-IV to CST-I	1.01 (0.48, 2.12)	1.46 (0.27, 7.77)
CST-IV to CST-III	0.90 (0.66, 1.21)	0.73 (0.46, 1.17)
Age at Sexual Debut 17+ vs. <17	N=4,397	
CST-I to CST-III	1.14 (0.78, 1.64)	0.84 (0.54, 1.31)
CST-I to CST-IV	0.68 (0.41, 1.13)	1.13 (0.63, 2.03)
CST-III to CST-I	0.97 (0.68, 1.37)	0.90 (0.58, 1.41)
CST-III to CST-IV	1.03 (0.79, 1.35)	0.94 (0.67, 1.33)
CST-IV to CST-I	1.62 (0.62, 4.20)	2.15 (0.47, 9.89)
CST-IV to CST-III	0.85 (0.65, 1.11)	0.81 (0.55, 1.20)
Antibiotics taken in past 30 days	N=4,401	
CST-I to CST-III	0.91 (0.60, 1.38)	1.00 (0.61, 1.64)
CST-I to CST-IV	1.61 (0.95, 2.71)	1.56 (0.89, 2.73)
CST-III to CST-I	0.98 (0.65, 1.46)	1.23 (0.83, 1.84)
CST-III to CST-IV	0.92 (0.68, 1.25)	0.97 (0.68, 1.36)
CST-IV to CST-I	1.01 (0.43, 2.39)	0.75 (0.33, 1.69)
CST-IV to CST-III	0.99 (0.74, 1.32)	1.09 (0.79, 1.50)

Bolded figures represent associations for which the 95% confidence interval does not include 1.

* Models are simultaneously adjusted for: Baseline SES, HSV-2 status, age, randomization status (intervention or control), age at sexual debut, and time-varying sexually active, SES, HSV-2 status, and antibiotic use in the past 30 days.

**Table 3.** Sub-Group Analyses: Effects of Menstrual Cups by Randomization Status

Stratum Specific Analyses: Effect of Intervention…	Crude Hazard Ratio (95% CI)	Adjusted Hazard Ratio (95% CI)^1^
Among those BV negative at Baseline	N=3,967	N=3,594
CST-I to CST-III	0.80 (0.58, 1.12)	0.93 (0.65, 1.34)
CST-I to CST-IV	0.64 (0.37, 1.09)	**0.48 (0.27, 0.85)**
CST-III to CST-I	1.09 (0.77, 1.53)	0.95 (0.66, 1.37)
CST-III to CST-IV	**1.32 (1.00, 1.73)**	**1.52 (1.12, 2.07)**
CST-IV to CST-I	0.70 (0.31, 1.60)	0.89 (0.40, 2.00)
CST-IV to CST-III	**1.29 (0.96, 1.72)**	**1.28 (0.93, 1.77)**
Among those Sexually active at baseline	N=1,569	N=1,461
CST-I to CST-III	0.72 (0.35, 1.48)	0.85 (0.17, 4.41)
CST-I to CST-IV	**0.47 (0.22, 1.00)**	0.88 (0.18, 4.21)
CST-III to CST-I	1.10 (0.61, 1.97)	1.00 (0.46, 2.17)
CST-III to CST-IV	**1.54 (0.97, 2.42)**	1.19 (0.69, 2.07)
CST-IV to CST-I	0.22 (0.01, 4.95)	0.94 (0.24, 3.66)
CST-IV to CST-III	1.21 (0.79, 1.86)	0.95 (0.59, 1.53)
Among those Not Sexually active at baseline	N=2,841	N=2,594
CST-I to CST-III	0.82 (0.57, 1.19)	0.93 (0.61, 1.42)
CST-I to CST-IV	0.68 (0.34, 1.35)	0.90 (0.46, 1.76)
CST-III to CST-I	0.99 (0.66, 1.50)	1.05 (0.66, 1.66)
CST-III to CST-IV	1.26 (0.91, 1.75)	1.08 (0.75, 1.54)
CST-IV to CST-I	1.37 (0.59, 3.16)	1.16 (0.55, 2.47)
CST-IV to CST-III	**1.37 (0.97, 1.93)**	1.25 (0.86, 1.82)
Among those STI Negative at Baseline	N=4,024	N=3,646
CST-I to CST-III	0.80 (0.57, 1.13)	0.89 (0.59, 1.34)
CST-I to CST-IV	**0.58 (0.35, 0.98)**	0.87 (0.50, 1.52)
CST-III to CST-I	0.92 (0.65, 1.30)	1.00 (0.66, 1.49)
CST-III to CST-IV	**1.34 (1.01, 1.76)**	1.11 (0.82, 1.52)
CST-IV to CST-I	1.08 (0.51, 2.31)	1.10 (0.55, 2.19)
CST-IV to CST-III	**1.35 (1.01, 1.80)**	1.18 (0.88, 1.59)
Among those HSV-2 Negative at Baseline	N=3,769	N=3,661
CST-I to CST-III	0.81 (0.57, 1.16)	0.95 (0.66, 1.38)
CST-I to CST-IV	0.62 (0.36, 1.07)	**0.50 (0.29, 0.85)**
CST-III to CST-I	1.10 (0.77, 1.56)	0.96 (0.65, 1.42)
CST-III to CST-IV	1.35 (1.02, 1.79)	**1.50 (1.11, 2.02)**
CST-IV to CST-I	0.84 (0.32, 2.21)	1.08 (0.41, 2.80)
CST-IV to CST-III	1.35 (1.01, 1.79)	**1.32 (0.97, 1.81)**
Among those HSV-2 Positive at Baseline	N=632	N=608
CST-I to CST-III	0.82 (0.36, 1.87)	0.89 (0.38, 2.09)
CST-I to CST-IV	0.84 (0.09, 8.02)	0.96 (0.21, 4.42)
CST-III to CST-I	0.89 (0.28, 2.87)	0.92 (0.00, >100)
CST-III to CST-IV	1.19 (0.61, 2.32)	1.09 (0.50, 2.40)
CST-IV to CST-I	0.70 (0.19, 2.52)	0.89 (0.00, >100)
CST-IV to CST-III	1.05 (0.49, 2.26)	0.94 (0.13, 6.83)
Among those with age at Sexual debut <17 years	N=1,487	N=1,368
CST-I to CST-III	0.89 (0.46, 1.72)	0.89 (0.21, 3.75)
CST-I to CST-IV	0.49 (0.23, 1.06)	0.86 (0.17, 4.47)
CST-III to CST-I	1.40 (0.79, 2.48)	1.25 (0.66, 2.39)
CST-III to CST-IV	1.28 (0.82, 2.00)	1.13 (0.63, 2.04)
CST-IV to CST-I	0.46 (0.05, 3.91)	1.01 (0.25, 4.06)
CST-IV to CST-III	1.13 (0.73, 1.74)	0.94 (0.59, 1.52)
Among those with age at Sexual debut >=17 years	N=2,910	N=2,673
CST-I to CST-III	0.86 (0.58, 1.27)	0.90 (0.56, 1.45)
CST-I to CST-IV	0.63 (0.30, 1.33)	0.91 (0.45, 1.85)
CST-III to CST-I	0.90 (0.58, 1.40)	0.94 (0.56, 1.56)
CST-III to CST-IV	**1.50 (1.08, 2.06)**	1.16 (0.81, 1.65)
CST-IV to CST-I	1.09 (0.49, 2.45)	1.09 (0.52, 2.27)
CST-IV to CST-III	**1.51 (1.07, 2.13)**	1.20 (0.84, 1.70)

Bolded figures represent associations for which the 95% confidence interval does not include 1.

1 Stratified models among BV Negative and HSV-2 Negative observations are simultaneously adjusted for age at baseline, time-varying SES, sexual activity, recent antibiotic use, age at sexual debut; BV model is additionally adjusted for time-varying HSV-2 status. For improved convergence, other models have varying co-variate adjustment and constraints (Supplemental Methods).

## Data Availability

This study was conducted with approval from the Kenya Medical Research Institute (KEMRI) Scientific and Ethics Review Unit (SERU), which requires that de-identified data from any Kenya-based study be released only after receipt of written KEMRI SERU approval for additional analyses. In accordance, trial data will be available upon request, after obtaining written KEMRI SERU approval for the proposed analysis. Application forms and guidelines can be accessed at https://www.kemri.org/seru-overview or by contacting seru@kemri.org.
