# Supplementary material for: Timing of menstrual cups to prevent transition from optimal to not optimal vaginal microbiome community state type: Results from a 6.5-year prospective observational cohort
Source: Res Sq. 2026 Jun 30:rs.3.rs-10177740. Preprint. [Version 1] doi: 10.21203/rs.3.rs-10177740/v1 (PMC13345564; doi:10.21203/rs.3.rs-10177740/v1)
Supplement: 1 [file NIHPPRS10177740V1-supplement-1.pdf]

Supplemental Statistical Methods

We applied a continuous-time Markov State model to estimate the probability of transitioning from one CST to another. Model fitting was conducted using the `msm` package in R (version 4.3.2),[20] with convergence achieved through iterative tuning of hyperparameters: maximum iterations (>5000 up to 20,000), relative tolerance thresholds (<1e-3 to 1e-8), and function scaling (10 to 4,000). To ensure model stability, in some stratum-specific adjusted analyses a reduced subset of the covariates was adjusted for, and CST transitions were constrained to share the same intensities for selected strata and adjusted covariates, and they are iterated here.

- Stratified models among BV Negative and HSV-2 Negative observations are simultaneously adjusted for age at baseline, time-varying SES, sexual activity, recent antibiotic use, age at sexual debut; BV model is additionally adjusted for time-varying HSV-2 status.
- Stratified model among sexually active: adjusted for age at baseline, time-varying SES, time-varying HSV-2 status, and age at sexual debut. Adjustment for recent antibiotic use leads to non-convergence. *Constraint*: For age at baseline, time-varying SES, and age at sexual debut, assume effects of transitioning from higher to lower CST (CST-IV to CST-III, CST-III to CST-I, or CST-IV to CST-I) are the same, and from lower to higher CST (CST-I to CST-III or CST-IV, and CST-III to CST-IV) are the same.
- Stratified model among not sexually active: adjusted for age at baseline, time-varying SES, time-varying HSV-2 status, and age at sexual debut.
- Stratified model among STI negative: adjusted for age at baseline, time-varying SES, time-varying HSV-2 status, age at sexual debut, and recent antibiotic use. Adjustment for sexually active leads to non-convergence.

- Stratified model among HSV-2 seropositive: adjusted for age at baseline, time-varying SES, sexual activity, age at sexual debut, and recent antibiotic use. *Constraint*: For all adjusted covariates, assume effects of transitioning from higher to lower CST are the same, and from lower to higher CST are the same.

Stratified models by age at sexual debut (<17 and >=17): adjusted for age at baseline, time-varying SES, sexual activity, time-varying HSV-2, and recent antibiotic use. *Constraint*: For age at baseline, time-varying SES, and sexual activity, assume effects of transitioning from higher to lower CST are the same, and from lower to higher CST are the same.

Supplemental Table 1. Results of Markov Modeling: Unadjusted and Multivariable Adjusted Hazard Ratios of Covariates on CST transitions

Covariates	Unadjusted HR (95% CI)	Adjusted HR[1] (95% CI)	Adjusted HR[2] (95% CI)
Engaged in transactional sex	N=4,406	N=4,364	N=4,041
CST-I to CST-III	0.67 (0.29, 1.52)	0.59 (0.26, 1.36)	0.69 (0.30, 1.61)
CST-I to CST-IV	**1.85 (0.93, 3.66)**	1.50 (0.76, 2.96)	1.28 (0.62, 2.63)
CST-III to CST-I	0.67 (0.34, 1.34)	0.69 (0.34, 1.41)	0.78 (0.39, 1.56)
CST-III to CST-IV	1.23 (0.82, 1.85)	1.12 (0.74, 1.68)	1.14 (0.73, 1.79)
CST-IV to CST-I	0.85 (0.25, 2.91)	1.30 (0.48, 3.53)	1.25 (0.43, 3.66)
CST-IV to CST-III	1.05 (0.70, 1.58)	0.98 (0.65, 1.48)	1.00 (0.64, 1.55)
Hormonal contraceptive use	N=4,411	N=4,364	N=4,041
CST-I to CST-III	1.40 (0.61, 3.21)	1.23 (0.51, 2.95)	1.76 (0.69, 4.46)
CST-I to CST-IV	**2.60 (1.15, 5.88)**	**2.24 (1.01, 4.98)**	1.36 (0.44, 4.17)
CST-III to CST-I	0.95 (0.53, 1.73)	1.13 (0.58, 2.20)	1.26 (0.57, 2.78)
CST-III to CST-IV	**1.30 (0.90, 1.88)**	1.12 (0.77, 1.65)	1.15 (0.76, 1.75)
CST-IV to CST-I	0.28 (0.03, 2.32)	0.24 (0.01, 4.79)	0.19 (0.00, 88.5)
CST-IV to CST-III	1.00 (0.72, 1.38)	0.99 (0.69, 1.42)	0.96 (0.62, 1.49)
Injectable contraceptive	N=4,441	N=4,364	N=4,041
CST-I to CST-III	2.13 (0.67, 6.79)	**2.79 (1.17, 6.65)**	**3.11 (1.37, 7.07)**
CST-I to CST-IV	1.58 (0.18, 13.9)	0.11 (0.00, >100)	0.04 (0.00, >100)
CST-III to CST-I	1.18 (0.54, 2.59)	1.36 (0.63, 2.96)	1.47 (0.66, 3.27)
CST-III to CST-IV	1.28 (0.70, 2.33)	1.34 (0.78, 2.31)	1.30 (0.75, 2.27)
CST-IV to CST-I	0.22 (0.00, 14.3)	0.04 (0.00, >100)	0.03 (0.00, >100)
CST-IV to CST-III	1.16 (0.72, 1.86)	1.23 (0.76, 1.99)	1.30 (0.79, 2.13)
Implant contraceptive	N=4,441	N=4,364	N=4,041
CST-I to CST-III	0.37 (0.03, 4.86)	0.08 (0.00, 73.5)	0.11 (0.00, 9.13)
CST-I to CST-IV	**4.30 (2.08, 8.88)**	**5.45 (2.65, 11.2)**	**5.50 (2.48, 12.2)**
CST-III to CST-I	0.63 (0.25, 1.61)	0.87 (0.31, 2.43)	0.90 (0.38, 2.10)
CST-III to CST-IV	1.29 (0.81, 2.05)	0.97 (0.58, 1.62)	0.86 (0.51, 1.47)
CST-IV to CST-I	0.27 (0.02, 3.41)	0.29 (0.01, 7.16)	0.24 (0.02, 3.12)
CST-IV to CST-III	0.87 (0.58, 1.29)	0.82 (0.55, 1.24)	0.73 (0.48, 1.11)
HIV positive (vs. negative)	N=4,431	N=4,332	N=4,027
CST-I to CST-III	1.36 (0.11, 17.0)	1.57 (0.20, 12.6)	3.05 (0.30, 31.2)
CST-I to CST-IV	**9.06 (2.94, 27.9)**	**4.50 (1.23, 16.5)**	3.12 (0.49, 20.0)
CST-III to CST-I	1.55 (0.40, 5.95)	0.89 (0.17, 4.57)	1.79 (0.28, 11.6)
CST-III to CST-IV	0.61 (0.13, 2.84)	1.02 (0.45, 2.31)	0.99 (0.40, 2.45)
CST-IV to CST-I	0.88 (0.06, 12.63)	0.72 (0.06, 8.01)	0.74 (0.09, 6.36)
CST-IV to CST-III	0.69 (0.36, 1.30)	0.66 (0.34, 1.30)	0.58 (0.26, 1.27)
Currently pregnant	N=4,406	N=4,364	N=4,292
CST-I to CST-III	1.27 (0.27, 6.00)	1.81 (0.66, 4.99)	1.63 (0.57, 4.66)
CST-I to CST-IV	**4.72 (1.88, 11.9)**	2.36 (0.80, 7.01)	2.56 (0.96, 6.85)
CST-III to CST-I	0.96 (0.32, 2.91)	0.86 (0.29, 2.60)	0.90 (0.30, 2.71)
CST-III to CST-IV	**2.32 (1.31, 4.11)**	**2.19 (1.25, 3.82)**	**2.15 (1.24, 3.73)**
CST-IV to CST-I	0.81 (0.04, 16.56)	0.94 (0.12, 7.54)	0.72 (0.06, 8.52)
CST-IV to CST-III	1.50 (0.74, 3.06)	1.39 (0.70, 2.75)	1.43 (0.72, 2.82)

[1] Simultaneously adjusted for intervention status, age at baseline, time-varying SES, sexual activity
[2] Simultaneously adjusted for intervention status, age at baseline, time-varying SES, sexual activity, recent antibiotic use, age at sexual debut, and time-varying HSV-2. For model of current pregnancy in relation to CST transition, HSV-2 is removed to achieve convergence.

Supplemental Table 2. Factors associated with menstrual cup use among intervention arm and control arm participants: results of unadjusted mixed effects models

	Intervention Arm Participants[1]		Control Arm Participants[2]	
**Covariates**	**Crude PR (95% CI), p-value**	**Adjusted PR (95% CI), p-value**	**Crude PR (95% CI), p-value**	**Adjusted PR (95% CI), p-value**
Baseline Age in years	**1.04 (1.02 – 1.07), <0.01**	1.02 (0.99 – 1.06), 0.24	NE	NE
Baseline Socioeconomic status (lower vs. higher)	**1.14 (1.01 – 1.28), 0.03**	**1.13 (1.02 – 1.25), 0.02**	NE	NE
Baseline Sexually active	**1.25 (1.09 – 1.45), <0.01**	**1.21 (1.05 – 1.39), <0.01**	NE	NE
Baseline STI positive	1.35 (0.98 – 1.85), 0.07		NE	NE
Baseline Has a boyfriend	1.17 (0.99 – 1.39), 0.07		NE	NE
Baseline BV positive	**1.08 (1.06 – 1.10), <0.01**		NE	NE
Baseline HSV-2 seropositive	1.05 (0.82 – 1.34), 0.71		NE	NE
Time-varying Socioeconomic status (lower vs. higher)	1.10 (0.98 – 1.23), 0.09		**1.27 (1.18 – 1.36), <0.01**	**1.23 (1.07 – 1.41), <0.01**
Time-varying Has a boyfriend	1.13 (0.93 – 1.38), 0.21		**1.36 (1.26 – 1.46), <0.01**	
Age in years at 30-month visit	NE	NE	0.97 (0.82 – 1.15), 0.74	
STI positive cumulative through 30-month visit	NE	NE	**1.22 (1.10 – 1.36), <0.01**	
Time-varying HSV-2 seropositive	0.97 (0.81 – 1.17), 0.77		**1.25 (1.13 – 1.38), <0.01**	**1.19 (1.12 – 1.28), <0.01**
Time-varying BV positive	1.10 (0.98 – 1.22), 0.09		1.17 (0.98 – 1.39), 0.08	1.13 (1.00 – 1.29), 0.06
Time-varying Type of sexual activity				
Not sexually active	reference	reference	reference	reference
Sexually active (no report of transactional or coerced sex)	**1.11 (1.04 – 1.19), <0.01**	1.06 (1.00 – 1.13), 0.05	**1.87 (1.33 – 2.62), <0.01**	**1.85 (1.17 – 2.93), <0.01**
Transactional sex	0.98 (0.86 – 1.12), 0.79	0.88 (0.75 – 1.04), 0.13	**2.43 (1.66 – 3.56), <0.01**	**2.54 (1.75 – 3.69), <0.01**
Coerced sex	**1.20 (1.02 – 1.42), 0.03**	**1.15 (1.03 – 1.29), 0.02**	**1.75 (1.45 – 2.12), <0.01**	**1.61 (1.16 – 2.24), <0.01**
Transactional & coerced sex	1.13 (0.84 – 1.51), 0.42	1.01 (0.79 – 1.30), 0.94	**2.49 (1.73 – 3.59), <0.01**	**2.42 (2.06 – 2.83), <0.01**

All models are time-adjusted. NE = "Not Examined"; aPR = Adjusted Prevalence Ratio; 95% CI = 95% Confidence Interval.
STI = Sexually transmitted infection; HSV-2 = Herpes simplex virus type 2; BV = Bacterial vaginosis;
Empty cells denote factors that were not statistically significant and not included in the multivariable models.
Values that are significant at the p<0.05 level are bolded.
[1]N=1,871 of 211 individuals; Age at baseline is maintained in the model due to indication of confounding when removed.
[2]N=1,055 of 203 individuals; Restricted to study visits occurring at 36-78 months
